# ^18^F-FDG PET Maximum-Intensity Projections and Artificial Intelligence: A Win-Win Combination to Easily Measure Prognostic Biomarkers in DLBCL Patients

**DOI:** 10.2967/jnumed.121.263501

**Published:** 2022-12

**Authors:** Kibrom B. Girum, Louis Rebaud, Anne-Ségolène Cottereau, Michel Meignan, Jérôme Clerc, Laetitia Vercellino, Olivier Casasnovas, Franck Morschhauser, Catherine Thieblemont, Irène Buvat

**Affiliations:** 1 LITO Laboratory, U1288 Inserm, Institut Curie, University Paris-Saclay, Orsay, France;; 2 Research and Clinical Collaborations, Siemens Medical Solutions, Knoxville, Tennessee;; 3 Department of Nuclear Medicine, Cochin Hospital, AP-HP, Paris Descartes University, Paris, France;; 4 Lysa Imaging, Henri Mondor University Hospital, AP-HP, University Paris East, Créteil, France;; 5 Department of Nuclear Medicine, Saint-Louis Hospital, AP-HP, Paris, France;; 6 Department of Hematology, University Hospital of Dijon, Dijon, France;; 7 Department of Hematology, Claude Huriez Hospital, University Lille, EA 7365, Research Group on Injectable Forms and Associated Technologies, Lille, France; and; 8 Department of Hematology, Saint Louis Hospital, AP-HP, Paris, France

**Keywords:** artificial intelligence, DLBCL, ^18^F FDG PET/CT, dissemination, metabolic tumor volume

## Abstract

Total metabolic tumor volume (TMTV) and tumor dissemination (Dmax) calculated from baseline ^18^F-FDG PET/CT images are prognostic biomarkers in diffuse large B-cell lymphoma (DLBCL) patients. Yet, their automated calculation remains challenging. The purpose of this study was to investigate whether TMTV and Dmax features could be replaced by surrogate features automatically calculated using an artificial intelligence (AI) algorithm from only 2 maximum-intensity projections (MIPs) of the whole-body ^18^F-FDG PET images. **Methods:** Two cohorts of DLBCL patients from the REMARC (NCT01122472) and LNH073B (NCT00498043) trials were retrospectively analyzed. Experts delineated lymphoma lesions from the baseline whole-body ^18^F-FDG PET/CT images, from which TMTV and Dmax were measured. Coronal and sagittal MIP images and associated 2-dimensional reference lesion masks were calculated. An AI algorithm was trained on the REMARC MIP data to segment lymphoma regions. The AI algorithm was then used to estimate surrogate TMTV (sTMTV) and surrogate Dmax (sDmax) on both datasets. The ability of the original and surrogate TMTV and Dmax to stratify patients was compared. **Results:** Three hundred eighty-two patients (mean age ± SD, 62.1 y ± 13.4 y; 207 men) were evaluated. sTMTV was highly correlated with TMTV for REMARC and LNH073B datasets (Spearman *r* = 0.878 and 0.752, respectively), and so were sDmax and Dmax (*r* = 0.709 and 0.714, respectively). The hazard ratios for progression free survival of volume and MIP-based features derived using AI were similar, for example, TMTV: 11.24 (95% CI: 2.10–46.20), sTMTV: 11.81 (95% CI: 3.29–31.77), and Dmax: 9.0 (95% CI: 2.53–23.63), sDmax: 12.49 (95% CI: 3.42–34.50). **Conclusion:** Surrogate TMTV and Dmax calculated from only 2 PET MIP images are prognostic biomarkers in DLBCL patients and can be automatically estimated using an AI algorithm.

## Visual Abstract

**Figure:**
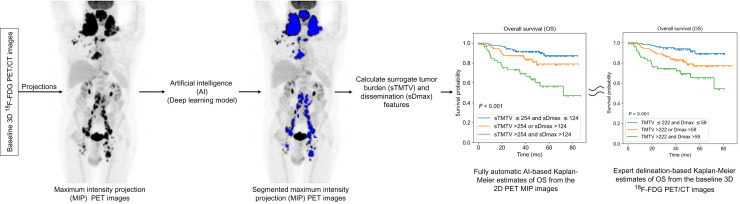

Diffuse large B-cell lymphoma (DLBCL) is the most common type of non-Hodgkin lymphoma. In clinical practice, ^18^F-FDG PET/CT is a standard of care for staging and assessing response in DLBCL patients (1). The prognostic value of the total metabolically active tumor volume (TMTV) measured from the whole-body ^18^F-FDG PET/CT images performed before treatment has been widely demonstrated in lymphoma, especially in DLBCL (2–6). The disease dissemination reflected by the largest distance between 2 lesions in the baseline whole-body ^18^F-FDG PET/CT image (Dmax) has been recently shown to be a complementary early prognostic factor compared with TMTV (7*,*^8^). TMTV and Dmax calculations require tumor volume delineation over the whole-body 3-dimensional (3D) ^18^F-FDG PET/CT images, which is prone to observer variability and complicates the use of these quantitative features in clinical routine.

To address this problem, automated lesion segmentation approaches using convolutional neural networks (CNNs) have been proposed (9*,*^10^). These methods require high computational resources to be developed but have shown promising results, despite missing small lesions (7). Results from CNN still need to be validated and adjusted by an expert before they are used for further analysis (7*,*^8^). This implies a thorough visual analysis of all 3D ^18^F-FDG PET/CT images and delineation of the lesions missed by the algorithm. Consequently, developing a pipeline that would speed up this checking/adjustment process is highly desirable in clinical practice.

Nuclear medicine physicians commonly use 2-dimensional (2D) PET maximum-intensity projection (MIP) views for visual interpretation as a synthetic representation of the 3D distribution of the tracer over the whole body. However, to the best of our knowledge, the prognostic value of PET parameters extracted from 2D MIP has never been explored. We hypothesized that tumor burden and spread could be automatically evaluated from only 2 PET MIP images corresponding to coronal and sagittal views. This would have 2 advantages: first, result checking and adjustment would be faster from 2 MIP views than the whole-body 3D ^18^F-FDG PET/CT images, typically including more than 200 transaxial slices. Second, a deep learning model for segmenting MIP images would involve fewer parameters than when segmenting the whole-body 3D ^18^F-FDG PET images. It is less computationally expensive and might require less data for training.

The purpose of this study was to investigate whether TMTV and Dmax biomarkers could be replaced by surrogate biomarkers automatically calculated using an artificial intelligence (AI) algorithm from only 2 MIPs of the whole-body ^18^F-FDG PET images. We then determined the prognostic values of the surrogate biomarkers in terms of progression-free survival (PFS) and overall survival (OS).

## MATERIALS AND METHODS

### Patient Cohorts

**FIGURE 1.**
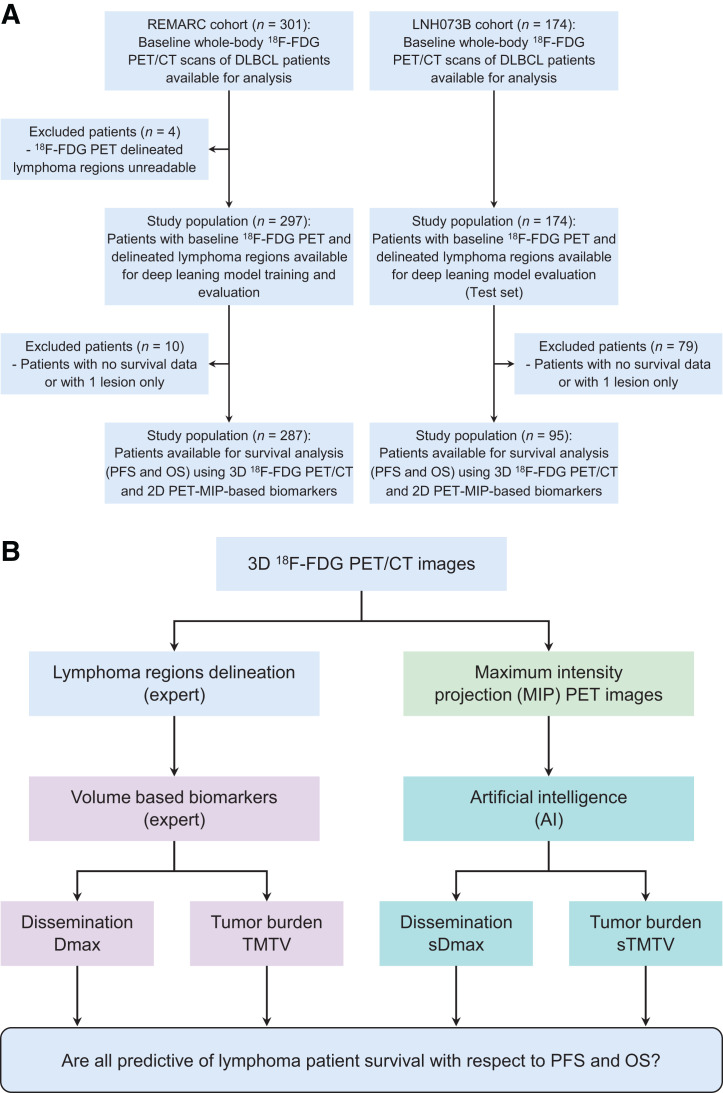
(A) Study flowchart. (B) Study design.

The study population included DLBCL patients who had undergone a baseline (before treatment initiation) PET/CT scan from 2 independent trials: REMARC (NCT01122472) and LNH073B (NCT00498043). The characteristics of these cohorts have been published elsewhere (6*,*^11^*,*^12^). PFS and OS as defined following the revised National Cancer Institute criteria (13) were recorded. Flow diagrams for the datasets and the study design are summarized in Figure 1. All data were pseudonymized before analysis. The institutional review board approval, including ancillary studies, was obtained for the 2 trials, and all patients provided written informed consent. The demographics and staging of the patients used for the survival analysis are summarized in Table 1.

**TABLE 1.** Population Characteristics

Characteristic	REMARC	LNH073B
No. of patients	287	95
Sex		
No. of men	165 (57.5%)	42 (44%)
No. of women	122 (42.5%)	53 (56%)
Median age (y)	68 (IQR, 64.0–73.0)	46 (IQR, 33.25–55.0)
Median weight (kg)	72 (IQR, 63.0–84.2)	68 (IQR, 58.0–80.0)
Median height (cm)	167.5 (IQR, 160.0–175.0) (1 case missed)	173 (IQR, 140.0–193.0)
Ann Arbor stage		
<I	1 (0.4%)	0 (0%)
≥II	286 (99.6%)	95 (100%)
Performance status		
0	115 (40%)	0 (0%)
1	121 (42%)	27 (28.4%)
2	42 (14.6%)	43 (45.3%)
3	2 (0.7%)	20 (21.1%)
4	2 (0.7%)	5 (5.3%)
Missing	5 (1.7%)	NA

IQR = interquartile range (quartile 1 to quartile 3); NA = not applicable.

### Measurements of Reference TMTV and Dmax

For the REMARC cohort, the lymphoma regions were identified in the 3D PET images as previously described (6*,*^14^), and the LNH073B lesions were segmented as previously explained (7). In all cohorts, physicians removed the regions corresponding to physiologic uptake and added pathologic regions missed by the algorithm. The supplemental materials (section A; supplemental materials are available at http://jnm.snmjournals.org) provide details. Expert-validated 3D lymphoma regions were used to compute the reference TMTV and Dmax (based on the centroid of the lymphoma regions), as shown in Figure 1B (8).

### Calculation of PET MIP Images and 2D Reference Lymphoma Regions

**FIGURE 2.**
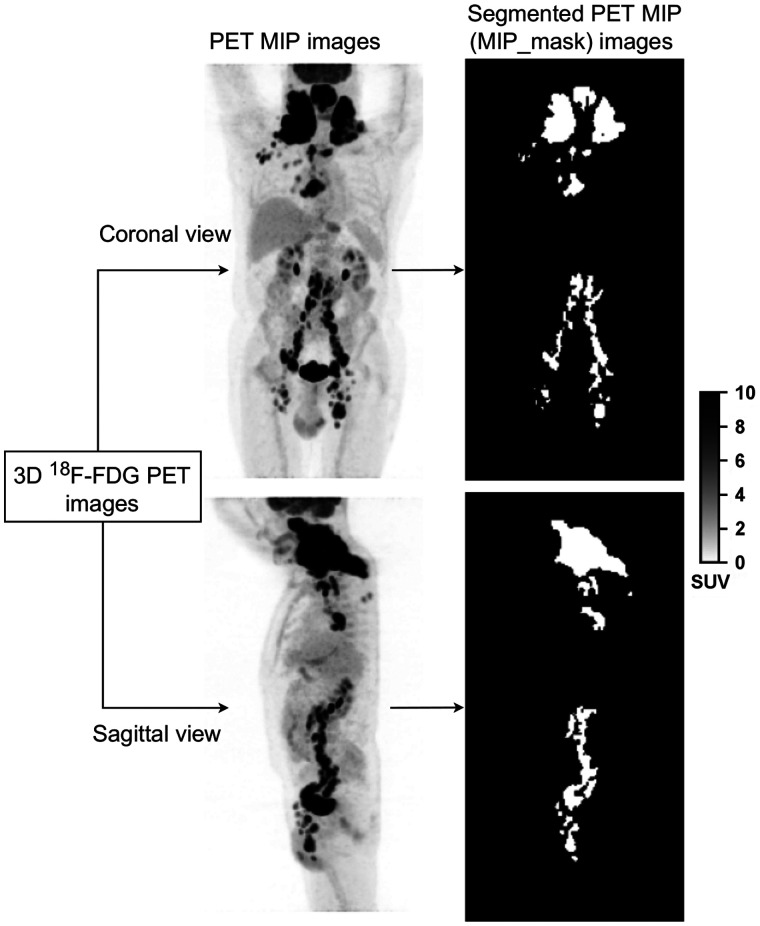
Example of ^18^F-FDG PET MIP images (left) and associated lymphoma regions (right) based on expert delineation of the 3D ^18^F-FDG PET images.

For each patient whole-body 3D ^18^F-FDG PET images and associated 3D lymphoma regions, two 2D MIP views and associated 2D lymphoma regions were calculated (Fig. 2). The 3D PET image was projected in the coronal and sagittal directions, 90° apart (Fig. 2), setting each pixel value of the projection to the maximum intensity observed along the ray normal to the plane of projection. Similarly, MIPs of the expert-validated 3D lymphoma regions were calculated, resulting in binary images of 2D lymphoma regions (Fig. 2), hereafter called MIP_ masks. As described in the following section, these MIP_masks were then used as a reference output to train a CNN-based fully automatic lymphoma segmentation method.

### Fully Automatic Lymphoma Segmentation on PET MIP Images

#### Deep Learning Model Inputs and Architectures

To automatically segment the lymphoma lesions from the sagittal and coronal PET MIP images, we adapted a previously published supervised 2D deep learning model (15). The sagittal and coronal PET MIPs were independent input images during training. The corresponding MIP_mask was the output image. The deep learning model was trained to transform a given sagittal or coronal PET MIP image to the corresponding MIP_ mask with pixels of lymphoma regions set to 1 and pixels of the nonlymphoma regions set to 0.

#### Training, Validation, and Testing Configurations

First, using the REMARC cohort (298 patients), a 5-fold cross-validation technique was used to train and evaluate the model. Patients were randomly split into 5 groups, and then 5 models were trained on 80% of the population and the remaining 20% was used for validation. The detailed network architecture (15*,*^16^) and the training procedures are fully described in the supplemental materials (section B; Supplemental Fig. 1) (17), following the CLAIM guidelines (18) and Society of Nuclear Medicine and Molecular Imaging AI Task force recommendations (19). The deep learning model is publicly available at https://github.com/KibromBerihu/ai4elife.

Second, we tested the model trained from the REMARC cohort (298 patients) on the independent LNH073B cohort (174 patients) to characterize its generalizability and robustness. The REMARC and LNH073B cohorts were acquired from 2 different trials. The REMARC study was a double-blind, international, multicenter, randomized phase III study, which started inclusion in 2010. In contrast, the LNH073B study was a prospective multicenter, randomized phase II study that started including patients in 2007.

### Calculation of Surrogate TMTV (sTMTV) and Surrogate Dmax (sDmax)

The sTMTV and sDmax were defined and computed from the MIP_ masks automatically segmented from the coronal and sagittal PET MIP images using the deep learning method.

#### Tumor Burden Analysis

To characterize tumor burden, we defined a surrogate tumor volume sTMTV from the MIP_mask as the number of pixels belonging to the tumor regions in MIP_mask multiplied by the pixel area. For a given patient, sTMTV was calculated from the coronal and the sagittal MIP_masks as sTMTV = sTMTV_coronal_ + sTMTV_sagittal_.

#### Tumor Dissemination Analysis

The spread of the disease was analyzed by estimating the largest distance between the tumor pixels belonging to the MIP_mask, using a new robust largest distance estimation approach. First, we separately calculated the sum of pixels along the columns and the rows of MIP_mask, yielding x and y profiles (Supplemental Fig. 2). Second, in each of these 2 profiles, the distances between the 2% percentile and the 98% percentiles (x_2%_ and x_98%_ in the x profiles, y_2%_ and y_98%_ in the y profiles) were calculated, yielding $\left({\text{x}}_{98\text{\%}}-{\text{x}}_{2\text{\%}}\right)$ and $\left({\text{y}}_{98\text{\%}}-{\text{y}}_{2\text{\%}}\right)$, respectively. These percentiles were chosen to improve the robustness of the calculation to outliers. The largest distance was defined as ${\text{sDmax}}_{\text{sagittal/coronal}}=\left({\text{x}}_{98\text{\%}}-{\text{x}}_{2\text{\%}}\right)+\left({\text{y}}_{98\text{\%}}-{\text{y}}_{2\text{\%}}\right)$. For a given patient, the surrogate tumor dissemination sDmax was the sum of the coronal and sagittal disseminations using ${\text{sDmax}=\text{sDmax}}_{\text{sagittal}}{+\text{sDmax}}_{\text{coronal}}$.

### Statistical Analysis

Using the MIP_masks obtained from the expert-delineated 3D lymphoma regions (Fig. 2) as a reference, the segmentation performance of CNN was evaluated using the Dice score, sensitivity, and specificity. The difference between the CNN-based segmentation results and the expert-delineated 3D lymphoma regions was quantified using Wilcoxon statistical tests. Univariate and multivariate survival analyses were performed. For all biomarkers, we calculated a time-dependent area under the receiver operating characteristics curve (AUC) (20). Bootstrap resampling analysis was performed to associate CIs to the Cox model hazard ratio (HR) and the time-dependent AUC (supplemental materials, section C, provide details). Test results were considered statistically significant if the 2-sided *P* value was less than 0.05.

## RESULTS

A total of 475 patients from 2 different cohorts were included in this study, of which 93 patients were excluded from the biomarker and survival analysis because the provided baseline ^18^F-FDG PET/CT images were not suitable to analyze all biomarkers (no PET segmentation by an expert or less than 2 lesions). Summary statistics of patients are presented in Table 1.

### Lymphoma Segmentation

**FIGURE 3.**
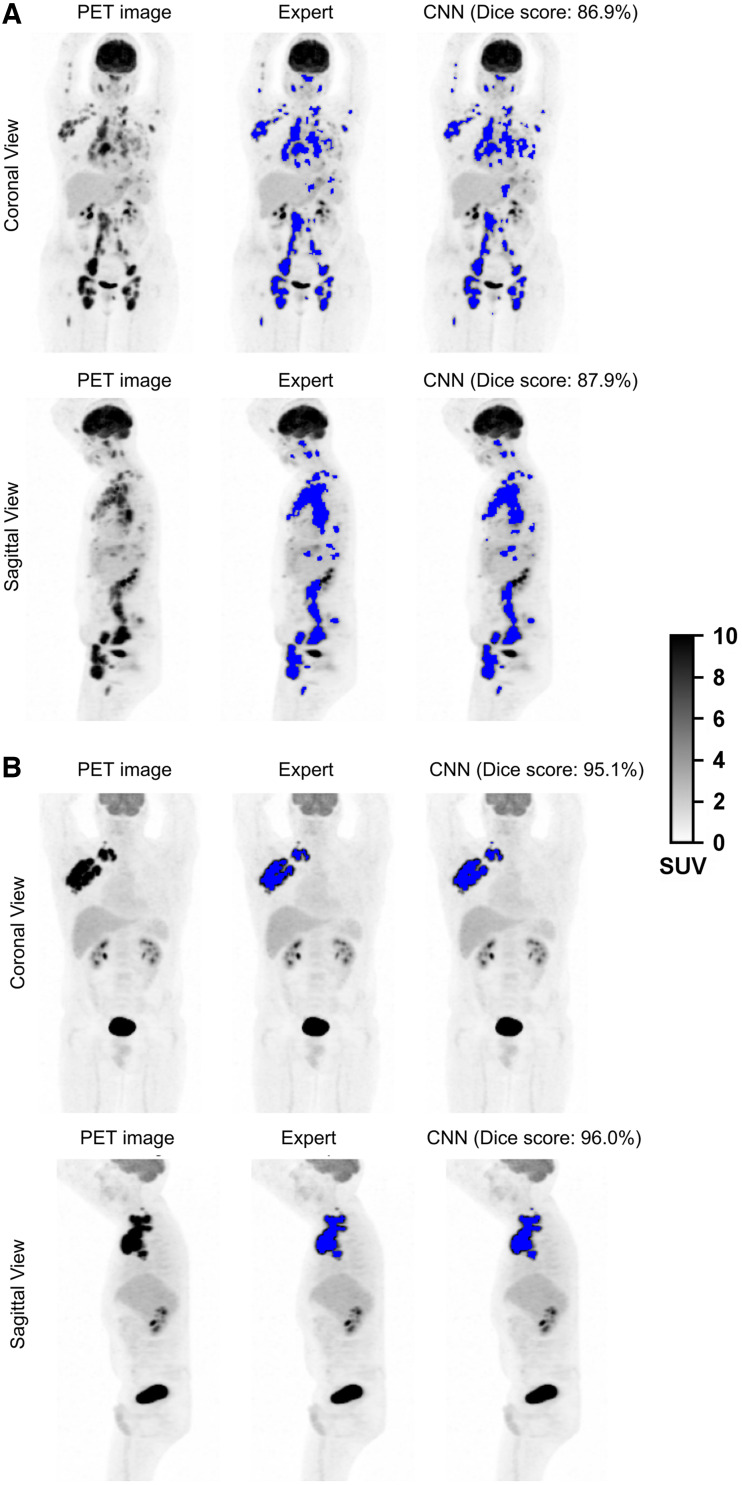
^18^F-FDG PET MIP images and segmentation results (blue color overlapped over PET MIP images) by experts (MIP_masks) and by CNN for 4 patients: from REMARC cohort (A) and from LNH073B cohort (B).

The performance of the proposed segmentation method was evaluated patientwise. The CNN segmentation method achieved a 0.80 median Dice score (interquartile range [IQR]: 0.63–0.89), 80.7% (IQR: 64.5%–91.3%) sensitivity, and 99.7% (IQR: 99.4%–0.99.9%) specificity for the REMARC cohort. On the testing set composed of 174 LNH073B patients, the CNN yielded a 0.86 median Dice score (IQR: 0.77–0.92), 87.9% (IQR: 74.9.0%–94.4%) sensitivity, and 99.7% (IQR: 99.4%–99.8%) specificity. In the LNH073B data, the CNN yielded a mean Dice score of 0.80 ± 0.17 (mean ± SD) on the coronal view and 0.79 ± 0.17 on the sagittal view. Figure 3 shows segmentation result examples from experts (MIP_masks) and CNN (Supplemental Fig. 3 provides more segmentation results). The Dice score was not significantly different (*P* > 0.05) between the coronal and sagittal views, both for the REMARC and the LNH073B cohorts.

In both cohorts, there was a significant correlation between ranked TMTV and Dmax values and the associated surrogate values obtained using CNN. For REMARC, TMTV was correlated with sTMTV (Spearman *r* = 0.878, *P* < 0.001), and Dmax was correlated with sDmax (*r* = 0.709, *P* < 0.001). Of 144 patients who had TMTV greater than the median TMTV (242 cm^3^), 121 (84.02%) patients had also sTMTV greater than the median sTMTV (174.24 cm^2^). One hundred forty-four patients had Dmax greater than the median Dmax (44.8 cm), and 113 (78.5%) of these patients also had sDmax greater than the median sDmax (98.0 cm).

For LNH073B, TMTV was correlated with sTMTV (*r* = 0.752, *P* < 0.001), and Dmax was correlated with sDmax (*r* = 0.714, *P* < 0.001). Of 48 patients who had TMTV greater than the median TMTV (375 cm^3^), 42 (87.5%) patients had also sTMTV greater than the median sTMTV (307.2 cm^2^). Forty-eight patients had Dmax greater than the median Dmax (44.1 cm), and 39 (81.3%) of these patients also had sDmax greater than the median sDmax (116.4 cm). Table 2 shows the descriptive statistics for the surrogate PET features.

**TABLE 2.** Statistics for Surrogate TMTV and Surrogate Dmax

Cohort	sTMTV/sDmax	Mean	SD	Minimum	Q1 (25%)	Median	Q3 (75%)	Maximum
REMARC	sTMTV (cm^2^)	252.27	245.75	0.48	77.04	174.24	350.56	1339.36
	sDmax (cm)	100.16	49.89	0.40	66.20	98.0	135.0	225.20
LNH073B	sTMTV (cm^2^)	388.12	249.91	63.68	224.48	307.2	450.08	1186.24
	sDmax (cm)	121.82	41.10	43.20	92.00	116.40	145.60	222.40

Q1 = first quartile (25% percentile); Q3 = third quartile (75% percentile).

### Survival Analysis

The time-dependent AUC and HRs with 95% CI of the metabolic tumor volume and tumor spread are shown in Table 3 for the REMARC and LNH073B data. All PET features extracted from the baseline 3D ^18^F-FDG PET/CT images and using AI (sTMTV and sDmax) were significant prognosticators of the PFS and OS.

**TABLE 3.** Results of the Univariate Analyses for PFS and OS Using Time-Dependent AUC Analysis and Cox Models (HR)

			3D ^18^F-FDG PET/CT estimates		2D PET MIP estimates	
Data	PFS/OS	Metrics	TMTV	Dmax	sTMTV	sDmax
REMARC	PFS	AUC	0.67 (0.60–0.73)	0.65 (0.58–0.72)	0.65 (0.58–0.72)	0.68 (0.62–0.75)
		HR	11.24 (2.10–46.20)	9.0 (2.53–23.63)	11.81 (3.29–31.77)	12.49 (3.42–34.50)
	OS	AUC	0.67 (0.58–0.76)	0.62 (0.53–0.71)	0.67 (0.58–0.76)	0.68 (0.59–0.76)
		HR	16.43 (2.42–77.29)	8.60 (1.47–28.33)	22.14 (4.73–69.06)	22.79 (3.80–79.21)
LNH073B	PFS	AUC	0.62 (0.49–0.75)	0.56 (0.39–0.72)	0.66 (0.53–0.80)	0.58 (0.41–0.74)
		HR	13.79 (0.45–86.80)	32.83 (0.4–220.8)	9.24 (0.95–37.94)	16.79 (0.69–86.41)
	OS	AUC	0.65 (0.46–0.82)	0.51 (0.31–0.72)	0.64 (0.45–0.82)	0.50 (0.29–0.72)
		HR	64.30 (0.74–384.80)	49.21 (0.07–258.3)	14.17 (0.59–67.02)	20.39 (0.08–93.66)

**FIGURE 4.**
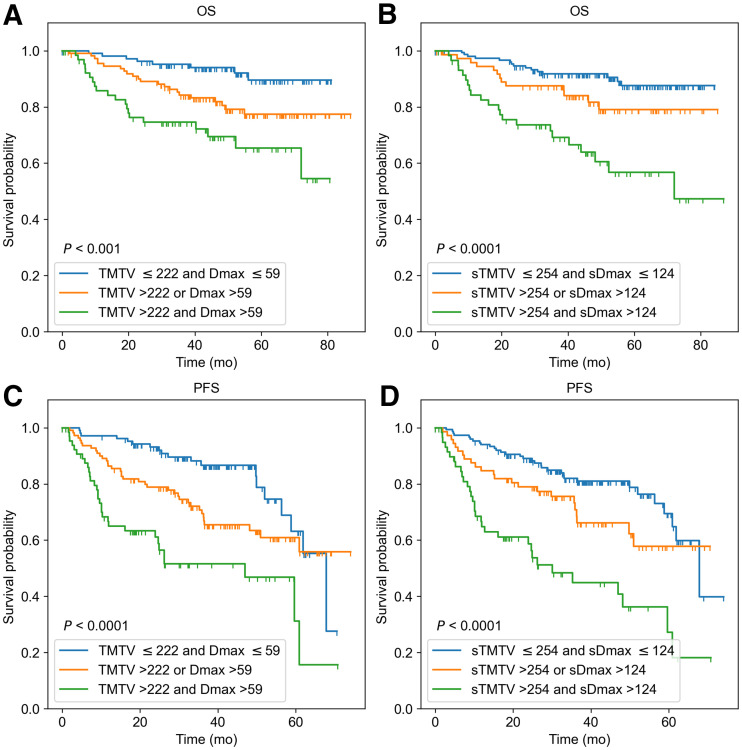
Kaplan–Meier estimates of OS and PFS from REMARC cohort according to 3D ^18^F-FDG PET/CT image–based features TMTV (cm^3^) and Dmax (cm) (A and C), and according to PET MIP image–based features sTMTV (cm^2^) and sDmax (cm) estimated from AI (B and D).

When TMTV and Dmax (or their surrogates) were combined, 3 risk categories could be differentiated in the REMARC data (Fig. 4): using the 3D features, category 1 corresponded to low TMTV (≤222 cm^3^) and low Dmax (≤59 cm) (low risk, *n* = 108); category 2 corresponded to either high Dmax or high TMTV (intermediate risk, *n* = 112); category 3 corresponded to both high Dmax and high TMTV (high risk, *n* = 67). This stratification was similar when using the MIP features–based categories using AI (Fig. 4). The accuracy of the CNN-based classification into 3 categories with respect to the 3D biomarkers–based classification was 71.4%.

**FIGURE 5.**
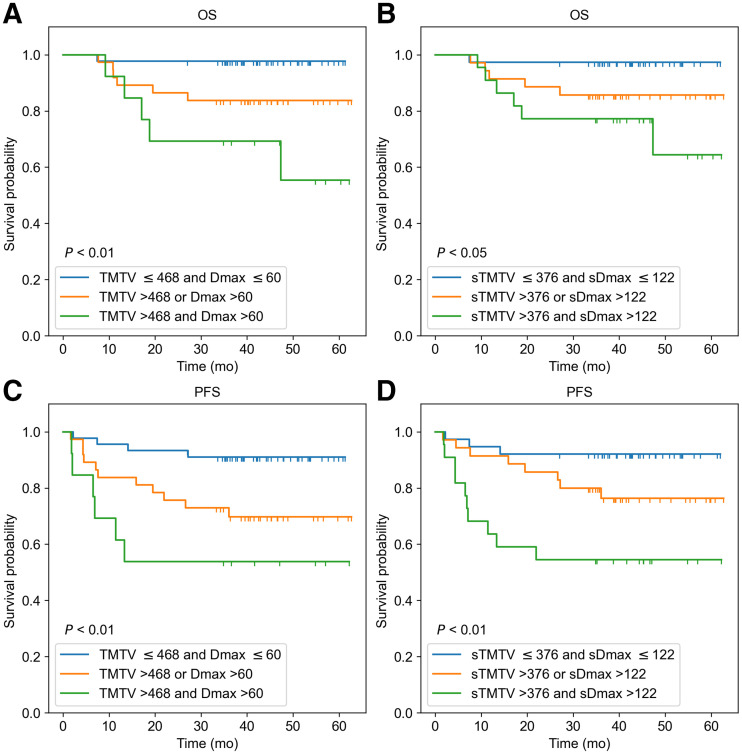
Kaplan–Meier estimates of OS and PFS from LNH073B cohort according to 3D ^18^F-FDG PET/CT image–based features TMTV (cm^3^) and Dmax (cm) (A and C), and according to PET MIP image–based features sTMTV (cm^2^) and sDmax (cm) estimated from AI (B and D).

In the LNH073B cohort, when TMTV and Dmax (or their surrogates) were combined, 3 risk categories could be differentiated (Fig. 5): using the 3D features, category 1 was defined as low TMTV (≤468 cm^3^) and low Dmax (≤60 cm) (*n* = 45); category 2 corresponded to either high Dmax or high TMTV (*n* = 37); category 3 corresponded to both high Dmax and high TMTV (*n* = 13). Of the 13 patients classified as high risk, 9 (69.2%) patients had less than 4 y of OS, and 10 (76.9%) patients had less than 4 y of PFS. This stratification was similar when using the CNN-based results. The sTMTV cutoff value was 376 cm^2^, and the sDmax cutoff value was 122 cm. There were 38 patients in category 1, 35 in category 2, and 22 in category 3. Of the 22 patients classified as a high risk, 19 (77.3%) patients had less than 4 y of OS, and 19 (86.4%) patients had less than 4 y of PFS. The accuracy of the AI-based classification into 3 categories with respect to the 3D biomarkers–based classification was 64.2%. All patients classified as high risk using the 3D biomarkers were also classified as high risk using the CNN, except 1 patient who had an OS of 36.6 mo. Of the 9 patients classified as high risk when using the CNN but not when using the 3D biomarkers, 8 (88.9%) patients had less than 4 y of OS, and the remaining 1 (11.1%) patient had 21.95 and 57.99 mo of PFS and OS, respectively.

In Supplemental Figure 4, the confusion matrices show the agreement between the 3D-based biomarkers and the surrogate MIP biomarkers in the LNH073B data. The percentage of the data classified into high, low, and intermediate risk is also shown. When a classification in 2 groups based on 1 biomarker only (either tumor burden or dissemination biomarkers) was used, the AI-based classification had a 79% accuracy compared with the 3D-based classification.

## DISCUSSION

We developed and evaluated a new framework to calculate sTMTV and sDmax (the largest distance between lymphoma sites) features from 2D PET MIP images. The motivation for considering tumor delineation on 2D MIP views instead of the 3D volume was 2-fold: first, checking lymphoma regions on 2D PET MIP images is much faster than on the 3D ^18^F-FDG PET/CT volumes. Second, training an automated AI tumor segmentation algorithm is easier in 2D than in 3D from a practical point of view (fewer parameters to be tuned, less data to be used for training, and less computational cost). We thus investigated the prognostic values of these surrogate biomarkers using 2 independent retrospective cohorts of DLBCL patients with baseline ^18^F-FDG PET/CT. Characterizing tumor burden and its dissemination was feasible using features extracted from the 2D PET MIP images. TMTV and Dmax were highly correlated with sTMTV and sDmax, respectively.

Developing automatic and robust lymphoma segmentation methods on PET MIP images could cost less data and less computational resources than when using the whole-body ^18^F-FDG PET images. It could allow AI experts to quickly investigate appropriate segmentation approaches to tackle the challenging lymphoma segmentation task and reduce intercenter and interexpert variations in lymphoma delineation. Experts can validate and correct, if necessary, AI results on 2D MIP images easier and faster than on their 3D volume counterparts. We also showed that a CNN could segment lymphoma lesions fully automatically from the given 2D PET MIP image with high accuracy compared with expert readers. This result was confirmed on the independent LNH073B cohort. The proposed CNN-based segmented regions enabled features extraction with predictive values comparable to when these features are calculated from the areas delineated by experts in the 3D image. The main strength of this work was that we validated our findings using an external cohort from a different retrospective trial. However, training the proposed deep learning model from an increased training sample size, preferably from different centers and acquisition parameters, might further improve its performance. No correlations were observed between the segmentation errors made by the model and lesion size. Previous lymphoma segmentation methods used the whole-body ^18^F-FDG PET/CT images (9*,*^10^). Most of these methods involved complex preprocessing, CT and PET image alignment, and did not investigate whether both TMTV and Dmax remained good prognosticators when calculated from the automated segmentation. Recent studies have also demonstrated that CNN-based results need corrections by experts (7*,*^8^). Correction of results on 3D volume could be time-consuming, observer-dependent, and difficult. In contrast, corrections, and validations (if necessary) could be easier and faster on 2D PET MIP images, allowing easy use of these features in clinical routine.

Interestingly, the surrogate biomarkers automatically calculated using AI (sTMTV and sDmax) had strong prognostic values regarding PFS and OS, comparable to the prognostic importance of TMTV and Dmax obtained from the 3D volumes. The classification of patients into the 3 risk groups using the 3D TMTV and Dmax agreed with the patient’s classification based on the 2D sTMTV and sDmax (71.4% and 64.2%, respectively, in REMARC and LNH037 cohorts). Patients classified as high-risk using 3D-based biomarkers and low-risk (or vice versa) using 2D-based biomarkers had values close to the cutoff values. Visual assessment of the segmentation results suggested that the 2D-based biomarkers tend to perform well compared with the 3D-based biomarkers when the patient had lesions spread over the body and performed less well when the patient had a large bulky lesion.

In this work, we defined and calculated the surrogate biomarkers from both the coronal and the sagittal PET MIPs. However, experiments showed that characterizing the lymphoma disease using sTMTV and sDmax calculated from either coronal or sagittal also had good predictive values, comparable to these features obtained from 3D volumes. The same cutoff values were used to analyze the PFS and OS. The cohorts were from 2 independent studies with varying stages of cancer (Table 1). Thus the (s)TMTV cutoff values were different between the 2 cohorts. Interestingly, the cutoff values to characterize the lesion dissemination (Dmax and sDmax) in DLBCL patients on baseline PET images were almost identical on the independent cohorts. Dmax and sDmax were defined empirically, yet a recent study has shown that the distance between lesions calculated using different distance measurement methods (namely Euclidean, Manhattan, and Chebyshev) in 3D yielded similar results in predictions of the outcome (21).

Our study has limitations. Although we validated the CNN on 2 independent retrospective studies, validating the proposed CNN in larger multicenter cohorts will be required to develop it into a clinical tool. In addition, although the CNN results can be easily visually checked, they should ideally be provided with a confidence level, which could be turned into a confidence associated with the risk classification.

## CONCLUSION

In this study, we introduced biomarkers extracted from PET MIPs as surrogates of the total metabolic tumor burden and tumor dissemination. To our knowledge, this is the first study showing that PET parameters extracted from 2D MIP images are predictive of outcome in a large series of patients with DLBCL, with results comparable to these features calculated from the 3D ^18^F-FDG PET/CT images. We demonstrated that surrogate TMTV and Dmax calculated from lymphoma regions automatically delineated on PET MIP images using AI have strong prognostic values in stratifying patients with DLBCL. This result might considerably facilitate the calculation and usage of these features in clinical practices.

## Notes

KEY POINTS **QUESTION:** Are surrogate tumor burden and dissemination features calculated from PET MIP images prognostic biomarkers in DLBCL patients and can they be automatically measured using an AI? **PERTINENT FINDINGS:** sTMTV and sDmax calculated from MIP of whole-body ^18^F-FDG PET images are predictive of PFS and OS in DLBCL patients from 2 independent cohorts. A CNN could segment lymphoma lesions from 2D PET MIP images automatically and the resulting CNN-based sTMTV and sDmax estimates were predictive of PFS and OS in 2 independent cohorts. **IMPLICATIONS FOR PATIENT CARE:** Surrogate tumor burden and dissemination features automatically calculated using AI from only 2 PET MIP images are prognostic biomarkers in DLBCL patients.
